# Fragment-Based Learning of Visual Object Categories in Non-Human Primates

**DOI:** 10.1371/journal.pone.0015444

**Published:** 2010-11-24

**Authors:** Sarah Kromrey, Matthew Maestri, Karin Hauffen, Evgeniy Bart, Jay Hegdé

**Affiliations:** 1 Brain and Behavior Discovery Institute, Medical College of Georgia, Augusta, Georgia, United States of America; 2 Vision Discovery Institute, Medical College of Georgia, Augusta, Georgia, United States of America; 3 Intelligent Systems Laboratory, Palo Alto Research Center (PARC), Palo Alto, California, United States of America; 4 Department of Ophthalmology, Medical College of Georgia, Augusta, Georgia, United States of America; Rutgers University, United States of America

## Abstract

When we perceive a visual object, we implicitly or explicitly associate it with an object category we know. Recent research has shown that the visual system can use local, informative image fragments of a given object, rather than the whole object, to classify it into a familiar category. We have previously reported, using human psychophysical studies, that when subjects learn new object categories using whole objects, they incidentally learn informative fragments, even when not required to do so. However, the neuronal mechanisms by which we acquire and use informative fragments, as well as category knowledge itself, have remained unclear. Here we describe the methods by which we adapted the relevant human psychophysical methods to awake, behaving monkeys and replicated key previous psychophysical results. This establishes awake, behaving monkeys as a useful system for future neurophysiological studies not only of informative fragments in particular, but also of object categorization and category learning in general.

## Introduction

Visual object perception is inextricably linked to object categorization. When we recognize an object, we classify it, however implicitly, into a known category. Various object recognition tasks, including detection, identification, and discrimination, are all categorization tasks of one type or another [1], [2], [3], [4], [5]. The neural mechanisms by which we categorize objects remain largely unclear (for overviews, see refs. [6], [7]).

Object categorization has been a challenging computational problem because it has not been clear how to handle the enormous range of image instances for a category (or, equivalently, class). For example, an instance of “human face” can be the image of any of an indefinite number of women, men, children, etc under a variety of viewing conditions (pose, illumination, size, etc). This raises the question of how to determine critical features for reliable categorization.

An important computational insight to emerge recently in this regard is that some features, or ‘fragments’, of the image can be objectively more informative about category membership than the image taken as a whole [5], [8], [9], [10], [11]. For instance, to distinguish the category of faces from another class, it can be more informative to use face fragments, such as the nose, lips, or an eye rather than to use the image of the whole face. In general, the fragments that are computationally most informative tend to be of intermediate complexity, which in this example corresponds to fragments of intermediate size relative to the object. This is because intermediate-complexity fragments tend to best balance category specificity against frequency of occurrence [5], [8], [9], [10], [11]. For instance, a fragment that includes more or less the whole face can reliably indicate the presence of a face in an image, but the chances of finding the exact same fragment in another image are relatively low. On the other hand, a small face fragment is more likely to appear in many different face images, but is also more likely to appear in non-face images, so that the fragment is not diagnostic of faces *per se* [5], [8], [9].

We have previously used human psychophysical studies to help understand how we learn and use informative fragments [12], [13]. To do this, we created novel, naturalistic categories of virtual 3-D objects using the virtual phylogenesis (VP) algorithm, which simulates biological processes of embryonic development and natural selection. We trained the subjects in novel categories using whole objects and tested the categorization performance using informative and uninformative fragments. We found that human subjects acquire informative fragments implicitly during the course of learning categories using whole objects.

Here we report that we have adapted these behavioral paradigms to macaque monkeys, and that the human psychophysical results are essentially replicable in monkeys. This report presents the monkey behavioral results so as to be as closely comparable to the previous human psychophysical study [12] (also see ref. [13]) as possible. Here we outline only the key experimental procedures and behavioral results. We will report the relevant neurophysiological results in a future report.

## Results

In order to adapt our previous human psychophysical paradigm [12], [13] to awake, behaving monkeys, especially in a form that facilitates future microelectrode recordings, we made four major modifications (see Materials and Methods for details): First, because monkeys will not work long without rewards, the animals were rewarded for their responses. Second, monkeys were required to categorize stimuli presented eccentrically while maintaining central fixation throughout the trial. Third, the stimuli were magnified as needed (but never shrunk) when presented at large eccentricities, so as to enable the animals to better resolve the stimuli. Finally, the stimuli were presented sequentially rather than simultaneously, so as to allow the temporal segregation of neural responses to various trial epochs.

### Creating Object Categories

**Figure 1:**
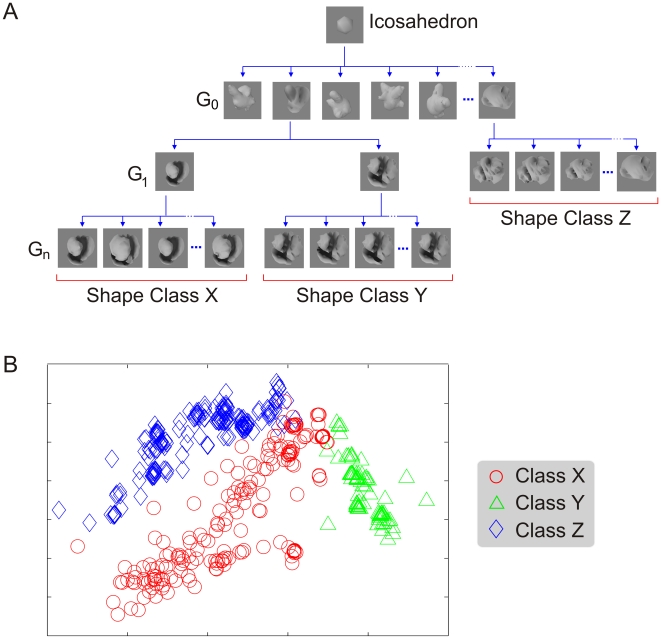
Naturalistic shape classes generated by virtual phylogenesis (VP). (**A**) The VP algorithm for generating naturalistic shape classes. This algorithm simulates biological evolution, in that shape characteristics evolve as random heritable variations are differentially propagated through successive generations [12], [13]. Note that the differences between, as well as within, the categories arise spontaneously and randomly during VP, rather than as a result of externally imposed rules, including the fragment selection process or any other classification scheme. The bottom of the evolutionary cascade denotes the three shape classes used in many of the experiments in this study. See refs. [12], [13] for additional examples of shape classes. (**B**) Shape variations within and across classes X, Y and Z as visualized by a metric multi-dimensional scaling (MDS) plot. Each data point represents one object from a given class (inset). MDS plots the data points so as to cluster similar data points together and disperse dissimilar data points from each other, so to provide a principled representation of the relevant classes (for details, see refs. [48], [49]).

We created several dozen naturalistic object classes using the VP [12], [13] algorithm described previously (see Fig. 1 and Materials and Methods for details). The categories were such that no two objects, including objects within a given category, were exactly alike. Therefore, distinguishing among them required learning the relevant statistical properties of the objects and ignoring the irrelevant variations.

The monkeys were trained and tested using multiple subsets of these classes. Consistent with our previous human psychophysical study [12], [13], the results were fundamentally similar regardless of the actual categories used (data not shown). Therefore, we present our results mainly using two representative subsets of three categories each.

### Isolating Informative Fragments

In Experiment 1, we used the three object classes, X, Y and Z, shown in Fig. 1. We isolated three different sets of 20 fragments each (see Materials and Methods for details). All three sets of fragments belonged to class X. The first set of 20 fragments (‘Main’ fragments) were each highly informative for Main task, defined as the binary classification task of distinguishing class X from class Y. The second and third set of 20 fragments each (‘Control’ fragments and ‘IPControl’ fragments) were uninformative for the Main task, but were visually comparable to the Main fragments. In addition, the IPControl fragments were visually interesting as defined by objective criteria, whereas the Control fragments did not have to meet these criteria. The mutual information (MI) value of a given fragment quantifies the amount of information it conveys about a given category [8], [9]. The higher the MI of a given fragment, the more useful the fragment is for categorization (*i.e*., the more information it conveys).

Fragments in Experiment 2 were isolated in an identical fashion, except that the Main task was to distinguish class Z from class X (see Materials and Methods for details).

### Testing the Informativeness of Individual Fragments

**Figure 2:**
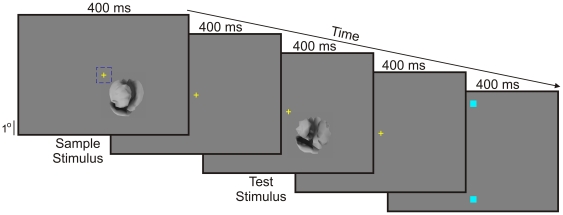
The trial paradigm. The animal performed a delayed same-different categorization task while maintaining fixation. After the animal established fixation on a central fixation target (‘+’) within an imaginary ±0.5° window (dashed square in far left frame), two eccentric stimuli were presented sequentially, each followed by a delay. After the second delay, the fixation target was turned off, at which time the animal indicated whether or not the two stimuli belonged to the same category by making a direct saccade to an appropriate saccade target (small blue squares in the far left frame). During the training phase of the study, both the stimuli during a given trial were whole objects. This figure shows a non-matching trial during training. Trials during the testing phase were identical (not shown), except that one of the stimuli during each trial was a fragment presented as a partial view of an object behind a light gray opaque occluder with a corresponding hole in it; the other stimulus in the trial was a whole object. In Experiments 1, 2 and 3, the fragment was presented as the first stimulus in each trial. See Materials and Methods for details.

**Figure 3:**
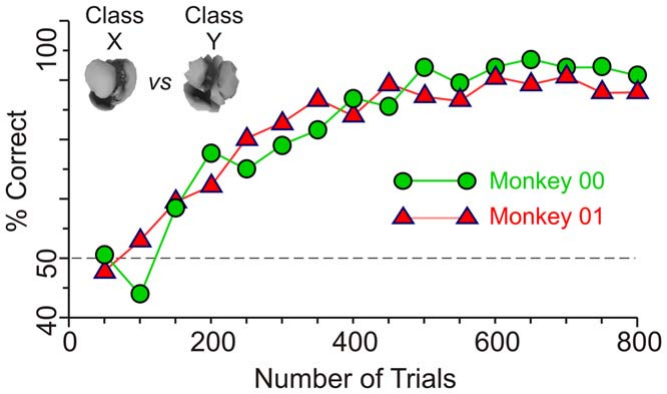
Changes in categorization performance as a function of training. Two animals fully trained in the categorization task learned two hitherto unknown categories (*icons at top left*). The percentages of correct trials during successive blocks of 50 trials each are shown for each animal. The *dashed line* represents chance level performance (50% correct). The training shown took 38 and 42 min from beginning to end, for Monkey 00 and Monkey 01, respectively. These learning durations mean that one can, in principle, carry out simultaneous microelectrode recordings of neuronal activity. See text for details.

The experiments consisted of training the monkeys using whole objects and then testing the animals using fragments, in both cases using a delayed same-different categorization task (Fig. 2; see Materials and Methods for details). Since only whole objects, not fragments, were used during training, the animals did not necessarily have to learn the fragments in order to learn the categories. Figure 3 shows the category learning curve of the two animals for class X *vs*. class Y (see inset). Note that at the start of the training, either animal performed at chance levels, indicating that the specific classes needed to be learned before the animals could classify the objects successfully. After several hundred trials, the performance of both animals was significantly above chance levels (binomial proportions test, *p*<0.05).

After the animals fully learned the categories, we tested the extent to which they were able to perform the classification task using only the fragments, each presented individually. We hypothesized, on the basis of the aforementioned computational considerations and our previous results in humans [12], [13], that if the animals unknowingly learned informative object fragments during the training of learning the whole objects, then the animals must be able to perform the categorization task using the individual Main fragments, but not the Control fragments.

**Figure 4:**
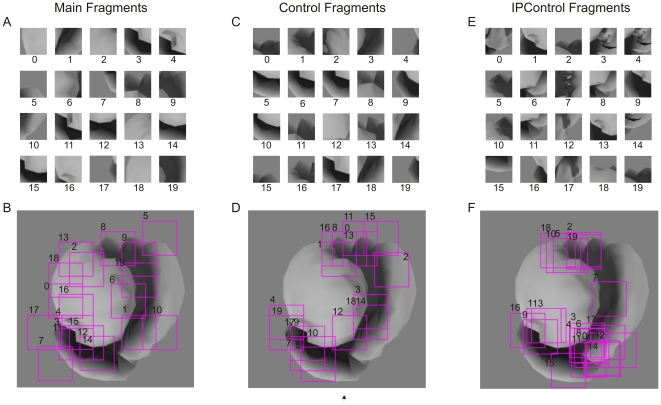
Object fragments used in Experiment 1. (**A**) Main fragments, which are 50×50 pixel fragments of objects from class X that are useful for distinguishing class X from class Y (Main task). (**B**) Location of the Main fragments, overlaid on a typical object from class X. Fragment borders are outlined in yellow for clarity. (**C**) Control fragments, which are fragments of objects from class X that are useful for distinguishing class X from class Z (‘Control task’), but are not useful for the Main task. (**D**) Location of the Control fragments. See text for details. (**E**) IPControl fragments, which were uninformative for the Main task, but comparable visually to the Main fragments. (**F**) Location of the IPControl fragments. In panel B, D and F, the fragments are overlaid on a typical object from class X. Note that the fragments were extracted from different images of class X, although they are overlaid together in this figure. Thus, although some of the fragments overlap in this figure, they are usually quite different in their visual content.

**Figure 5:**
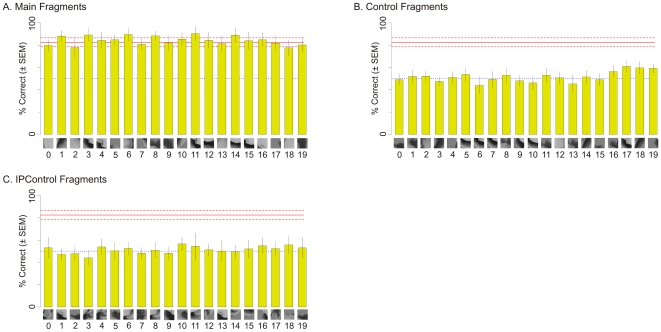
Performance of the animals in Experiment 1. Each bar shows the average percentage (±SEM) of trials in which the animals classified a given object as belonging to class X given the corresponding fragment. The *dotted blue line* denotes 50%, or chance level performance. The *light red lines* denote the mean (solid line) and ±SEM (dashed lines) performance of the animals during the respective last four blocks of training. Panels (**A**), (**B**) and (**C**) show the responses of the animals in the Main task (*i.e*., X *vs*. Y) using Main, Control and IPControl fragments respectively. With each Main fragment, the performance was significantly above chance (binomial tests, *p*<0.05), and indistinguishable from the performance using whole objects (binomial tests, *p*>0.05, data not shown).

The various fragments used in Experiment 1 are shown in Figure 4, and the MI values of the fragments are summarized in Table 1. The observed performance of the animals in this experiment closely matched the above predictions (Fig. 5). The animals performed the Main task significantly above chance using each of the Main fragments (Fig. 5A; binomial tests, *p*<0.05 for each fragment). Thus, the animals were able to categorize the objects based on each of the Main fragments by itself, consistent with the above hypothesis.

**Table 1:** Mutual Information of Individual Fragments in Experiment.

Fragment			MI		
Type	Belonged to Category	Categorization Task	Mean	SEM	Range
Main	X	X *vs*. Y (Main task)	1.0	0	1.0–1.0
Control	X	X *vs*. Z (Control task)	0.99	0	0.99–0.99
		X *vs*. Y (Main task)	0.33	0.022	0.17–0.48
IPControl	X	X *vs*. Y (Main task)	0.11	0.008	0.06–0.17

Moreover, the animals were unable to perform the Main task above chance levels using any of the Control or IPControl fragments (Fig. 5B and C, respectively; binomial tests, *p*>0.05). That is, the animals were about equally likely to classify an object as belonging to class X or class Y on the basis of a given Control or IPControl fragment. Therefore, although all three types of fragments belonged to class X, only the Main fragments, which contained informative information about the class, were likely to be assigned to class X.

**Figure 6:**
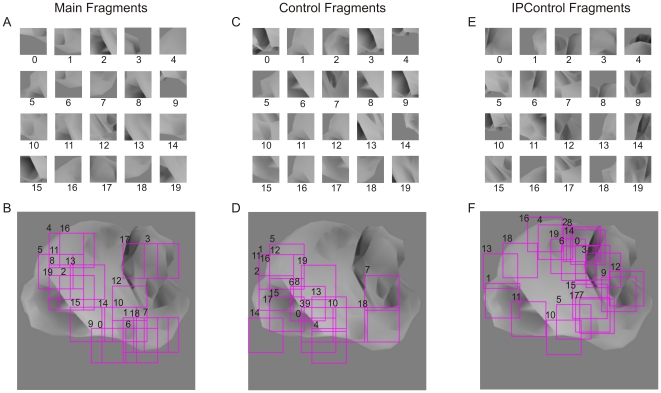
Object fragments used in Experiment 2. (**A**) Main fragments, which are 50×50 pixel fragments of objects from class Z that are useful for distinguishing class Z from class X (*i.e*., Main task in this experiment). (**B**) Location of the Main fragments, overlaid on a typical object from class Z. Fragment borders are outlined for clarity. (**C**) Control fragments, which are fragments of objects from class Z that are useful for distinguishing class Z from class Y (‘Control task’), but are not useful for the Main task. (**D**) Location of the Control fragments. See text for details. (**E**) IPControl fragments, which were uninformative for the Main task, but comparable visually to the Main fragments. (**F**) Location of the IPControl fragments. In panel B, D and F, the fragments are overlaid on a typical object from class Z. Note that the fragments were extracted from different images of class Z, although they are overlaid together in this figure. Thus, although some of the fragments overlap in this figure, they are usually quite different in their visual content.

**Figure 7:**
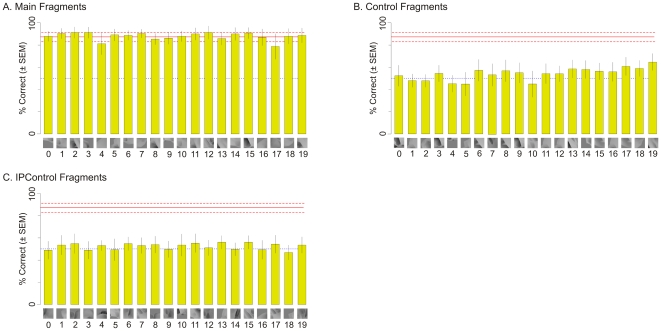
Performance in Experiment 2. Each bar shows the average performance (±SEM) of the animals with a given fragment. The *dotted blue line* denotes 50%, or chance level performance. The *light red lines* denote the mean (solid line) and ±SEM (dashed lines) performance of the animals during the respective last four blocks of training. Panels (**A**), (**B**) and (**C**) show the responses of the animals in the Main task *(i.e*., Z *vs*. X) using Main, Control and IPControl fragments respectively. With each Main fragment, the performance was significantly above chance (binomial tests, *p*<0.05), and indistinguishable from the performance using whole objects (binomial tests, *p*>0.05, data not shown).

To ensure that the above results were not attributable to a chance designation of object classes, we performed Experiment 2 in which we repeated the design of Experiment 1, but with a different set of classes, whereby the Main task was to distinguish class Z from class X (see Materials and Methods, Fig. 6, and Table 2). The same two monkeys participated in this experiment. The results of this experiment were similar to those in Experiment 1 (Fig. 7). Note that the results of these two experiments together essentially replicate in monkeys our previous human psychophysical results [12], [13].

**Table 2:** Mutual Information of Individual Fragments in Experiment 2.

Fragment			MI		
Type	Belonged to Category	Categorization Task	Mean	SEM	Range
Main	X	Z *vs*. X (Main task)	0.99	0	0.99–0.99
Control	X	Z *vs*. Y (Control task)	0.99	0	0.99–0.99
		Z *vs*. X (Main task)	0.61	0.025	0.24–0.68
IPControl	X	Z *vs*. X (Main task)	0.009	0.005	0.05–0.13

### Learning Fragments *vs*. Whole Objects

One distinction that was not addressed in our previous human studies [12], [13] is whether subjects actually learn fragments during training, or are simply able to access whole objects from fragment information. For instance, the subjects could have learned whole objects during training and, during the subsequent testing, could have performed the categorization task by simply matching the fragment to the most similar whole object.

The results of Experiments 1 and 2 indicate that this was not the case (Figs. 5 and 7). The MI of some Control fragments for the Main task was significantly higher than 0. In experiment 1, Control fragments 13 through 19 had MI of 0.4 or above (not shown). Similarly, in Experiment 2, Control fragments 3 through 19 had MI of 0.5 or above (not shown). While this is much lower than the MI of Main fragments in either experiment, it still allows for better-than-chance categorization. Therefore, if the animals learned whole objects, they would be able to access these objects from Control fragments as well and perform categorization with better-than-chance accuracy. The fact that they were unable to do so and utilize the available information suggests that this information was not learned during training, and that learning therefore was limited to fragments, as opposed to whole objects, in our case.

### Stability of the Categorization Performance During Training

**Figure 8:**
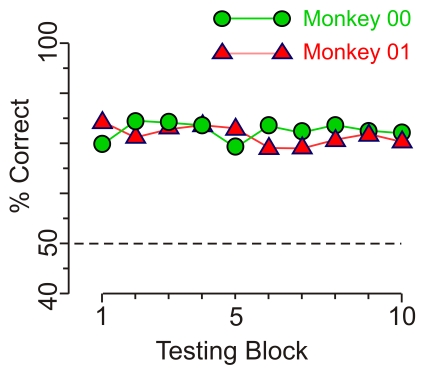
Performance over the course of testing in Experiment 2. The performance of the animals during the testing sessions is shown. Each data point is averaged from 120 trials from each animal (60 trials each of Main- and Control fragments), randomly interleaved with each other. Note that the performance is lower than the average response for Main fragments (Fig. 7A), because the responses were averaged across Main- and Control fragments. Similar results were obtained when the data were analyzed separately for Main-, Control- or IPControl fragments in this experiment and in Experiment 1 (3-way ANOVA, testing blocks x fragment number x fragment type; *p*<0.05 for testing blocks).

One potential concern about the above results is that the animals may have learned the fragments during the testing itself, because they were rewarded for correct responses. If this is the case, one would expect that (a) the performance would be at or near chance levels at the beginning of testing, since the animals had not encountered the fragments before, and (b) the performance would improve during the course of testing, as the animals presumably learned the fragments based on feedback implicit in the rewards. An examination of the testing data showed that neither of the above scenarios was applicable to our data (Fig. 8). The performance was above chance levels and stable throughout the testing (binomial proportions test, *p*>0.05). These results are consistent with a scenario where the animals learned the fragments to asymptotic levels during training with whole objects, and therefore rewarding them during testing did not result in any further learning.

We also found that similar stable, asymptotic performance during testing can be obtained with *random* rewarding, provided the testing blocks were relatively short (< = 60 trials) and were interspersed with the ‘refresher’ training blocks when the animal was rewarded non-randomly (data not shown). However, we found that testing with the above non-random reward regimen is preferable, since it elicited highly stable performance even when the testing blocks were up to 1000 trials long (data not shown).

### Efficacy of M-scaling

We enlarged all the stimuli (including fragments and whole objects) corresponding to their intended eccentricity to help compensate for the progressive reduction in visual acuity with increasing eccentricity (see Materials and Methods for details). M-scaling enlarges the stimulus by about 2.5 arcmin for every increase of eccentricity by 1°. Since whole objects were relatively large (∼4.5°) to begin with, the increase in size due to M-scaling was a relatively small fraction of the original size. Therefore, as expected, M-scaling of whole images or larger fragments (50×50 pixel fragments, which subtended 0.89°×0.89° without magnification) did not significantly affect the animals' performance (binomial proportions test, *p*>0.05; data not shown).

There are two main scenarios in which the stimuli may be smaller and/or less resolvable than those used in Experiments 1 and 2 above. First, depending on the given categories, the physical size of the most informative fragments may be smaller than the 50×50 pixel fragments used in the above experiments (see, *e.g.*, refs. [12], [13]; also see ref. [8]). Second, depending on the viewing distance and eccentricity, the fragments of a given physical size may have a smaller retinal size or larger eccentricity.

**Figure 9:**
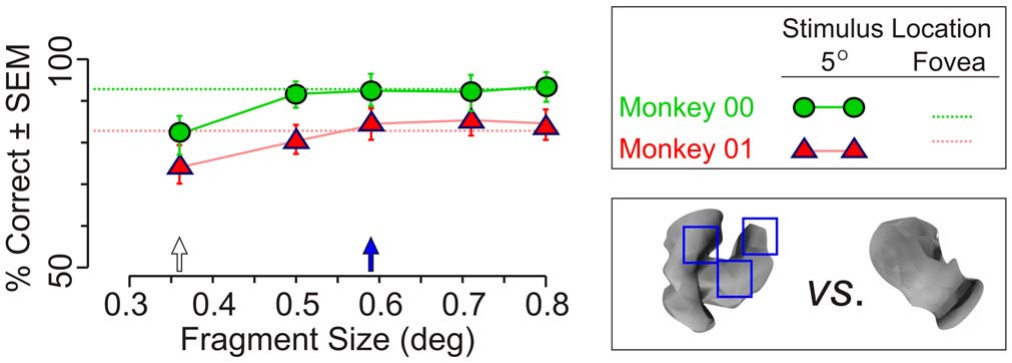
Effect of M-scaling on categorization performance in Experiment 3. The animals performed a fragment-based categorization task where the sample stimulus in each trial was a fragment and the test stimulus was a whole object. All stimuli were presented at an eccentricity of 5° in the lower right quadrant. The performance of the animals for Main fragments is shown as a function of the fragment size. The performance for the Control fragments was at chance levels, as expected (not shown). For any given size, all stimuli, including all whole objects and fragments, were magnified by the same scaling factor. See Materials and Methods for details. The *open arrow* denotes the original size of the fragment (*i.e*., without magnification). The *filled blue arrow* denotes the M-scaled size appropriate for 5°. The *dotted horizontal lines* denote the performance of either animal when the stimuli were viewed foveally at their original, unmagnified size. Typical objects from the Main class and Control class are shown in the bottom right inset, with three selected Main fragments highlighted by *blue squares* on the object from the Main class.

To determine whether M-scaling can have a measurable ameliorative effect in such cases, we carried out Experiment 3 using a different, randomly chosen trio of categories in which the informative fragments were 20×20 pixels (or 0.36°×0.36°; Fig. 9). We essentially repeated Experiments 1 and 2 using these fragments. The dotted lines in Figure 9 denote the average performance of either animal for the Main fragments when the animals carried out the task foveally. Consistent with the results of Experiments 1 and 2, this performance was statistically indistinguishable from the animals' performance using whole objects (not shown).

The green circles and red triangles in Fig. 9 denote the animals' performance when all stimuli were presented at an eccentricity of 5° at various magnifications (see legend for details). When the fragments were presented at their original, unmagnified size (open arrow on x-axis), the performance was significantly lower compared to the performance at the same size at the foveal location (filled circle/triangle *vs.* dotted lines; *t* tests, *p*<0.05). This indicates, as expected from previous studies [14], [15], [16], [17], [18], [19], [20], that the effects of increasing eccentricity are more pronounced for smaller stimuli. However, when the fragments were magnified, the performance at the eccentric location improved. When the fragments were presented at the magnification dictated by M-scaling (filled arrow on x-axis), the performance was indistinguishable from the performance elicited by unmagnified fragments presented at the fovea. Further magnifications did not improve the performance. Together, these results indicate, consistent with the findings of many previous studies [14], [15], [16], [17], [18], [19], [21], [22], that the effects of eccentric viewing are appreciable when the stimuli are relatively small, and that M-scaling offers a principled method for compensating for the decrease in acuity.

## Discussion

The present study is important in two main respects. First, it provides additional behavioral confirmation, in macaque monkeys, of our previous human psychophysical finding that informative fragments are learned during category learning. Second, it adapts the study of category learning and fragment-based categorization to macaque monkeys and makes it suitable for not only behavioral studies, but also future neurophysiological studies.

### Fragment-Learning as a Part of Category Learning

We have previously offered a detailed explanation of the implications of fragment-based category learning [12], [13]. We will therefore only briefly summarize the relevant arguments here.

Our results indicate that the category learning in monkeys is similar to humans, at least in that monkeys also learn informative, intermediate-complexity fragments as a matter of course when they learn new object categories, even though the animals were not explicitly required to learn the fragments. It was clearly not computationally necessary for the animals to learn the fragments, because the tasks could be performed based on whole objects. The performance of the animals was a function of the task-relevance of the fragments. Moreover, the animals did not consistently associate task-irrelevant fragments to correct learned categories, even when the fragments were otherwise visually interesting. Together, these results indicate that monkeys selectively learn informative fragments incidentally as a part of category learning. Moreover, as noted earlier, our results suggest that the animals learned fragments *per se* and did not use fragments to access the internal representations of whole objects. It is also worth noting that the animals were able to perform the categorization task using relatively brief stimulus duration (400 ms; see Materials and Methods), whereas human subjects in our previous study were allowed unlimited viewing time [12], [13].

In previous studies of novel category learning in humans and animals alike, the algorithm for generating novel objects depended on the algorithm for classifying them into categories [4], [6], [23], [24], whereas the two were independent in our case, as they are in nature. To the extent that our stimuli reflected natural categories, our results indicate that such incidental learning of fragments may be a common principle of learning of natural object categories in monkeys (see below).

### Implications for the Mechanisms of Category Learning

The neuronal mechanisms of fragment-based category learning have not been studied extensively in monkeys, although some neurophysiological studies have explored the neuronal mechanisms of category learning in general [7], [24]. The methods described in this report can help address this, because they are specifically designed to be amenable to future monkey neurophysiological studies.

Two previous human studies, Harel et al [25] and Lerner et al [10], have examined the extent to which informative fragments support categorization of objects into familiar categories. Both showed that the ability of subjects to decide whether a given fragment was a part of a familiar object (such as a car, a face or some other object, in case of Harel et al [25]) correlated with the MI of the fragment. As noted in the Introduction, our study differed from these previous studies in key respects, three of which are worth summarizing here. First, by using novel stimuli classes, we are able to study category learning, rather than just categorization. Second, since we controlled subject training, our fragments were extracted from the same set of images used by subjects during category learning. Third, the training images were completely unoccluded in our case and, moreover, the classes we used were linearly separable. This eliminates the possibility that the subjects could have learned the fragments out of necessity (*e.g*., to cope with occlusions).

Our result that fragment-learning accompanies category learning is significant, because it straightforwardly links category learning with categorization, in that informative fragments play a role in both. To the extent that informative fragments have potential neural correlates at intermediate levels of visual processing [5], [8], [10], [25] (also see ref. [26]), the same neural correlates may play a role in category learning. Thus, category learning may not necessarily be limited to the highest levels of visual processing (see refs. [7], [27], [28], [29], [30]).

### Usefulness of VP for Monkey Neurophysiological Studies of Category Learning and Categorization

Apart from the fact that the VP algorithm represents a novel method of creating object categories (*cf*., ‘Greebles’ [4], [31]), the resulting categories have several desirable features for the study of categorization and category learning. First, the categories have measurable but randomly arising within-class variations. In most of the earlier studies using object categories created by compositing shape primitives, there tends to be little or no within-class variation (for reviews, see refs. [4], [23], [32], [33]). But in natural scenes, two exemplars of a given category are seldom identical. The VP algorithm addresses this by generating naturalistic categories which mimic not only the shape variations of, but also the hierarchical relationships among, natural objects [1], [2], [34].

Second, note that although we used digital embryos as the substrate for VP in the present study, any virtual object, biological or otherwise, real-world or otherwise, can be used as VP substrates, and the algorithm can be readily modified to simulate more complex phylogenetic processes (*e.g*., convergent evolution, in which different taxa, such as whales and fish, come to resemble similar visual categories).

Third, VP can be used to generate a hierarchy of categories, directly analogous to the hierarchy of categories of biological objects in nature [1], [2], [34]. This means that VP can also be a useful tool for exploring our hierarchical understanding of natural objects.

Finally, if necessary, both within-class variants and between-class variants can be artificially selected to fit desired distributions. This means, on the one hand, that the categories can be generated based on, or independently of, an *a priori* classification algorithm, as desired. On the other hand, it provides a convenient tool for choosing categories such that the animals learn them within minutes, or over several months, so as to facilitate short term (*e.g*., using transdural electrodes) as well as long term studies (*e.g*., using chronic implants) of neural mechanisms of category learning.

### Methodological Adaptations for Monkey Neurophysiology

It is worth noting that all the key human psychophysical results were reproducible in monkeys despite the four major adaptations mentioned earlier: modifications in feedback (*i.e*., reward) regimen, eccentric stimulus presentation with central fixation, M-scaling, and delayed (rather than simultaneous) categorization. Of these, the latter three modifications were made with future neurophysiological studies in mind, where it is more likely than not that the recording locations will be parafoveal. When parafoveal viewing or neurophysiological recording is not contemplated, one or more of the modifications can be dispensed with as appropriate. Only the reward regimen needs to be necessarily different for monkeys.

It is worth emphasizing that M-scaling appears to be needed when the stimuli are relatively small. Previous studies have reported that the limit of foveal resolution in macaques is about 0.67 arcmin [22] (also see ref. [35] and the references therein). This means that as the foveal size of the intended stimuli begins to approach this size, M-scaling will be correspondingly more important. M-scaling is also advisable as a principled method for resolving the confounding effects of varying eccentricity and/or size when one needs to compare responses at more than one eccentricity or retinal size.

Given that the delayed same-different task in monkeys yielded essentially the same results as simultaneous match-to-sample task used in humans [12], [13], it seems reasonable to assume that the latter task would yield the similar results in monkeys. Such simultaneous stimulus presentation paradigms are especially useful when it is desirable to reduce the memory load [36], [37], [38].

Altogether, our results not only demonstrate the feasibility of carrying out research on fragment-based categorization in monkeys, but also suggest, although do not prove, that the category learning and categorization may be fundamentally similar in humans *vs.* monkeys.

## Materials and Methods

### Creating Naturalistic Object Classes Using VP

We created novel, naturalistic categories using the VP algorithm as described previously [12], [13]. Briefly, this algorithm simulates key processes of biological evolution, whereby shape variations among objects of a given generation arise randomly. All variations are heritable in principle, in that each object starts as an exact replica of its parent and develops further on its own. Selection is externally imposed, and consists of the fact that at each generation, only some of the objects are allowed to generate descendents. The children of a given parent constitute an object class (Fig. 1A). Thus, categories arise naturally in VP by means of selective propagation of heritable variations.

As input to VP, we used novel, naturalistic virtual 3-D objects called digital embryos [39]. Using VP, we created about 20 novel classes of objects (three of which are shown in Fig. 1), each containing up to 200 different objects. Note that the classes were generated without any regard to whether or how they could be classified and whether or not they contained any fragments useful for this classification. Each 3-D object was rendered without externally applied texture and with the same viewing and lighting parameters against a neutral gray background in the OpenGL graphics environment (www.opengl.org). The images were stored as 8-bit, 256×256 pixel grayscale bitmaps.

### Animal Subjects and Surgical Procedures

All animal-related procedures used in this study were approved in advance by the Medical College of Georgia Institutional Animal Care and Uses Committee (IACUC; Permit #08-08-102). All animal protocols fully conformed with the National Institutes of Health Guide for the Care and Use of Laboratory Animals and with the recommendations of the Weatherall Report.

Surgical procedures were carried out essentially as described before [40], [41], [42], [43]. Briefly, two adult male macaques (*Macaca mulatta*; 8–9 kg) were used in this study. Prior to behavioral training, each animal was implanted with a custom-made titanium head-post using titanium cranial screws (Gray Matter Research, Bozeman, MT) and an acrylic cranial patch (Palacos Bone Cement; Zimmer Inc., Warsaw, IN) using sterile surgical procedures. After the animals recovered from surgery, they were trained in the behavioral tasks described below.

### Experiments

We carried out several independent experiments using several independent subsets of the categories. As noted in the Results section, the experiments were largely similar, and differed mainly in terms of which category was distinguished from which, and yielded fundamentally similar results. For this reason, this report will describe the results of three representative experiments.

Experiments 1 and 2 were carried out using classes X, Y and Z shown in Fig. 1. Experiment 3, a smaller experiment in which we manipulated the stimulus size (see below), used classes L, M and N (class distributions not shown; see Fig. 9 for two exemplars). Each experiment was carried out in three phases: (i) extracting fragments, (ii) training the animals in the categories using whole objects, and (iii) testing the animals using fragments.

### Fragment Extraction

#### Extracting Fragments for Experiment 1

For this experiment, the ‘Main’ categorization task was defined as distinguishing objects of class X from objects of class Y. Twenty informative fragments supporting the Main task were isolated (‘Main’ fragments). Each Main fragment was a small 50×50 pixel (0.89°×0.89°) sub-image of a class X object.

The fragments were isolated using the same procedure as described in our previous human psychophysical experiments [12], [13]. Briefly, each class consisted of 200 different images, corresponding to 200 different objects. Out of a practical necessity to limit the size of the search space for finding informative fragments, a smaller, randomly chosen subset of 20 images from each class was used as input to the fragment isolation algorithm. All 50×50 pixel fragments on a dense grid (with step size of 15 pixels) were considered for each image. This resulted in several hundred candidate fragments for each of the 20 images. MI of each fragment for the Main task was calculated. The fragment with the highest MI was selected, and the set of candidate fragments was pruned based on visual similarity (see below) to this selected fragment. The process was repeated until a total of 20 Main fragments (Fig. 4A) were selected.

Visual similarity was evaluated using the correlation coefficient of pixel values. To detect small overlaps between fragments, the correlated fragments were also allowed to move with respect to each other. Candidate fragments with visual similarity above 0.8 were considered too similar to a previously selected fragment and were removed. This constraint reduced shape redundancy across the selected fragments.

Main fragments are useful for performing the Main task. We therefore expected the animals to preferentially use these fragments during this task. To assess the degree of this preference, non-informative fragments need to be selected as a basis for comparison.

A naive approach would be to select fragments as above, but with minimal, rather than maximal, MI. A disadvantage of this approach is that it tends to select visually uninteresting fragments. For example, image patches that are uniform or almost uniform in intensity have very low MI, so that several of these would typically be selected by the naive approach. Such fragments would indeed be uninformative, but for a trivial reason. To make the comparison fair, it is desirable to avoid selecting such fragments.

As in the previous human psychophysical study [12], [13], we used two principled methods of selecting interesting but uninformative fragments for comparison. First, we introduced a ‘Control’ task, which is to discriminate class X from class Z. Twenty fragments that are uninformative for the Main task were selected from the same aforementioned subset of 20 images, subject to the constraint that the fragments have high MI for the Control task (‘Control’ fragments). As before, these were selected from a pool of candidate fragments – all 50×50 pixel fragments of a class X object on a dense grid. First, all candidate fragments with MI for the Control task less than 0.7 were removed (recall that the MI can vary between 0 and 1 in our case). Next, fragments uninformative for the Main task were selected as described above, but with minimal (instead of maximal) MI. The intuition behind this method is that visually uninteresting fragments are expected to be uninformative for any task. The constraint of having high Control task MI therefore ruled out such fragments. Indeed, the resulting Control fragments (Fig. 4C) have significant visual content.

We also isolated 20 additional fragments using an interest point detector (‘IPControl’ fragments) from the aforementioned subset of 20 images. Interest point detectors select areas of an image that have significant visual content, such as corners or intersections [44] or high entropy [45]. Such detectors are heavily used in computer vision (for a review, see ref. [46]). In our experiments, we used the popular Harris interest point detector [44], [47]. First, we detect all interest points in an image. Since these points are by definition visually interesting, we then simply proceed to select 20 fragments with low MI for the Main task (as before, subject to the constraint of being visually dissimilar to one another) (see Fig. 4E).

Compared to Control fragments, IPControl fragments explore the set of uninformative fragments more fully, since the criterion for selection is based more directly on local visual content. By contrast, Control fragments are constrained to be informative for an auxiliary task (the Control task), and this criterion will certainly miss those visually interesting fragments which happen to be uninformative for the Control task. On the other hand, the IPControl fragments may be uninformative for a trivial reason. Interest point detector rules out the most trivial cases (such as patches of uniform intensity), but may still pass other uninteresting content (for example, a patch containing high spatial frequency random noise). Control fragments do not run that risk since they are guaranteed to be informative for some other task (the Control task) and therefore are useful for categorization.

To summarize, we selected a total of 60 fragments for Experiment 1. All of these are sub-images of the Main class objects. Out of these fragments, 20 are informative for the Main task, and 40 are uninformative.

#### Extracting Fragments for Experiment 2

The goal of our experiments was to determine whether monkeys learn to use informative fragments in categorization. However, Experiment 1, described above, only involves a single categorization task (the Main task). To ensure the results are not specific to this particular combination of categories, it is desirable to evaluate performance on a different combination of categories. In Experiment 2, we used the same three object classes (X, Y, and Z), but redefined their roles. To this end, the Main task was designated as distinguishing objects of class Z from objects of class X, and the Control task was designated as distinguishing class Z from class Y. We then selected 60 additional fragments using the procedure described above, but using the new class designations (see Fig. 6).

#### Extracting Fragments for Experiment 3

The same procedures as above were used to extract smaller, 20×20 pixel (or 0.36°×0.36°) fragments from a new trio of categories, L, M and N. The Main task was to distinguish class L from class M, and the Control task was to distinguish class L from class N.

### Training in the Categories

Prior to learning any of the novel categories described in this report, the animals were fully trained in the categorization task itself using unrelated sets of categories. Thus, in each given experiment, the animals learned new categories using the previously learned task paradigm, and not the task paradigm *per se*.

The task consisted of delayed same-different categorization as shown in Figure 2. The animal categorized stimuli presented at an eccentricity of 0°–8° (depending on the experiment, see below) while maintaining fixation. Eye position was monitored using a video eye tracker (Arrington Research, Scottsdale, AZ). For Experiments 1 and 2, all stimuli were presented at an eccentricity of 8° during both training and testing phases of the experiment. For Experiment 3, stimuli were presented both foveally (0°) or at 5° during both phases of the experiment. The performance was generally much worse when the testing was carried out at a retinal location different from the trained location (data not shown). For parafoveal stimulus presentations, the stimuli were suitably enlarged so as to compensate for the decrease in visual acuity with increasing eccentricity (see below).

During each trial, the animal had to establish fixation within 200 ms of the fixation spot onset and maintain fixation within a ±0.5° window until the fixation spot was turned off toward the end of the trial (see below). Two hundred milliseconds after the animal had established fixation, a sample stimulus was presented for 400 ms followed by a 400 ms delay, following which a test stimulus was presented for 400 ms. Following another 400 ms delay, the fixation spot was turned off, at which time the animal had to report whether or not the sample- and the test stimuli belonged to the same category by making a direct saccade to a designated target. The animal received a drop of liquid food reward for all and only the correct responses, and an audible beep for incorrect responses. The next trial started after a variable 1–4 s inter-trial interval.

In a given experiment, monkeys were trained in the Main task appropriate for that experiment. For instance, in Experiment 1, the sample stimulus was drawn randomly from class X or class Y. The test stimulus was drawn randomly from class X for matching trials, and from class Y for non-matching trials. The matching *vs*. non-matching trials occurred in the equal proportions in randomly interleaved order over the course of a block of trials.

A given animal was considered trained if it performed significantly above 75% correct (*i.e*., at *p*<0.002 by binomial test) and the performance remained asymptotic (as determined by one-way ANOVA at *p*>0.05) for at least four consecutive blocks of 50 trials each. Depending on the classes, the animals learned the classes to this criterion within an hour (Fig. 3) or over several weeks (not shown). Until the end of a given experiment, the animals received daily ‘refresher’ training using the same training paradigm as before so as to maintain asymptotic performance.

During the entire experiment, the animals received no training in the Control task, nor shown any object from the third class (class Z in Experiment 1, class Y in Experiment 2). The reason is that all fragments used in a given experiment were evaluated only with respect to MI in the Main task, while the Control task played only an auxiliary role.

The training procedure for Experiments 2 and 3 was identical to that for Experiment 1, except that the class designations were different, as described above.

### Enlarging the Stimuli for Peripheral Viewing (M-scaling)

In all experiments, we enlarged the stimuli (without changing their pixel resolution) according to the stimulus eccentricity using the well-established M-scaling method [14], [15], [16], [17], [18], [19], [21], [22]. Briefly, previous studies have shown that if a stimulus is magnified at peripheral locations in proportion to M^−1^, where M is the cortical magnification factor, the stimulus becomes equally resolvable across the visual field. This scaling, referred to as M-scaling, is known to be the same for macaque monkeys as well as humans [14], [15], [16], [17], [18], [19], [21], [22]. For eccentricities up to 30°, enlarging the stimulus by 2.5 arcmin per degree of eccentricity adequately compensates for reduction in acuity caused by increasing eccentricity (ref. [21], p. 589). While this scaling had negligible effect on the performance using whole objects (presumably because the proportional increase in size was negligible), the performance did benefit from M-scaling when stimuli were relatively small (see, *e.g.*, Fig. 9). Nonetheless, we M-scaled all stimuli in all experiments. In any given experiment, all stimuli, including fragments as well as whole objects, were scaled by the same scaling factor, determined by their intended eccentricity [14], [15], [16], [17], [18], [19], [21], [22]. We emphasize that we always enlarged (and never shrank) the stimuli without resampling, so that the information in the stimulus was unaffected by the scaling.

The M-scaling method has small, known shortcomings [14], [15], [17], [18], [19]. However, our results indicate that this method is adequate for our purposes, in that the animals' performance at the foveal location is statistically indistinguishable from their performance at peripheral locations when the images are M-scaled (see, e.g., Fig. 9).

### Testing the Fragments

Trials during the testing phase of the experiment were identical to the trials during the training phase, except as noted otherwise. During the testing phase, the sample- and the test stimuli were always a fragment and a whole object, respectively. As before, the animal had to report whether the two stimuli were drawn from the same class. The sample stimulus was generated by compositing the fragment of interest with a light gray occluder with a corresponding hole in it.

The trials for the various Main-, Control-, and IPControl fragments were randomly interleaved. For each fragment, the performance of each animal was measured over four blocks of 60 trials each spread over two or more days.

### Data Analysis

Data were analyzed using scripts custom-written in Matlab (Mathworks, Natick, MA) or R (r-project.org). Only the data in which the animal maintained fixation throughout the required period were further analyzed. Since it was not feasible to test multiple subjects as in our previous human psychophysical studies, we instead averaged the test data over a larger number of repetitions (240 trials per fragment, except where noted otherwise) from each of the two animals.
